# “I can’t get it into my head that I have cancer…”—A qualitative interview study on needs of patients with lung cancer

**DOI:** 10.1371/journal.pone.0216778

**Published:** 2019-05-14

**Authors:** Henrikje Stanze, Nils Schneider, Friedemann Nauck, Gabriella Marx

**Affiliations:** 1 Department of Palliative Medicine, University Medical Center Göttingen, Göttingen, Germany; 2 Institute for General Practice, Hannover Medical School, Hannover, Germany; 3 Department of General Practice / Primary Care, University Medical Center Hamburg-Eppendorf, Hamburg, Germany; University of Auckland, NEW ZEALAND

## Abstract

**Background:**

Caring for patients with advanced lung cancer is of high relevance in different clinical settings. Lung cancer is among the most common causes of death from malignant neoplasms worldwide; with increasing prevalence and mortality.

**Aim:**

To get a better understanding of individual patients’ needs, exploring the experiences and meaning of living with advanced lung cancer at the end of life, and to develop strategies for improving patient-centred care in Germany.

**Design:**

Qualitative explorative interview study with patients, using grounded theory.

**Setting/Participants:**

A sample of 17 adults living with advanced lung cancer in Lower Saxony/Germany was recruited in two university hospitals. Patients were asked to tell of their experiences of living with advanced lung cancer. The emphasis of this study was the period of palliative tumour therapy.

**Results:**

The main phenomenon of living with advanced lung cancer is the feeling of having to redefine one’s own existence, such as social roles within and outside the family. The diagnosis trigger powerlessness, which can lead to information passivity, followed by acceptance of aggressive tumour treatment. Patients perceive a high degree of psychological and social stress, without being able to express this. There is a lack of regular appropriate psychosocial care accompanying chemotherapy. Patients ascribe their physical suffering to the side effects of tumour treatment, which may trigger a desire to die. Finally, patients tend to hide their individual needs, even when asked.

**Conclusions:**

Regarding the patients’ needs, greater emphasis must be placed on psychosocial care as part of the biopsychosocial model to adequately consider the patients’ concerns. Assessments can be helpful to enhance communication at an early stage across all professions into the multi-professional therapy.

## Introduction

Malignant growths in the lung are among the most common causes of death from cancerous diseases in Germany and worldwide [1,2], with an increasing tendency. Since lung cancer is symptomless for a long time and the first diagnosis, small-cell or non-small-cell, is often made at a very advanced stage (IIIB and IV) [3–7], tumour treatment with primary palliative goals often starts immediately after diagnosis. In terms of the incidence of non-small cell to small cell lung carcinoma, the ratio is about 80% to 20%, but both have a low relative five-year survival rate and are two of the tumour entities with the worst prognoses [1,2,7]. Although carcinomas of the lungs have different degrees of degeneration, the general symptom burden can be similar, due to the respiratory tract and bronchial infestations. Based on this circumstance, no differentiation was made between the carcinoma types of the lungs in the selection of participants recruited for this study.

Recent studies show that people with inoperable lung cancer experience more physical burden than those diagnosed with other cancers [4,8]. These symptoms include physical weakness, fatigue syndrome, pain, loss of appetite, insomnia and respiratory distress [4,6–11], usually associated with significant limitations in daily life. Furthermore, lung cancer patients report more psychological distress than people with other cancers [12]. Integrated specialised palliative care, e.g. alongside the tumour-specific treatment, focusing on the individual needs of patients with advanced lung cancer, can improve quality of life [11, 13–16]. The German S3 guideline distinguishes between needs (subjective, individual demands), normative needs (objectively recognisable), and resources (“material means” and “possibility of a person to deal with a difficult experience”) [15–17], and recommends focusing on the patients’ individual needs, which were addressed in this study.

Although there have been many quantitative studies conducted on end-of-life issues with lung cancer [4,6,7,9,10], hardly any qualitatively empirical studies exist on experiences and meaning of living with advanced lung cancer at the end of life from the patients’ view. This knowledge makes it possible to derive specific, illness-related individual and unconscious needs. Although the scoping literature review by Ramsenthaler et al. focused on crises with lung cancer (in particular breathlessness), patients’ perspectives and experiences, and their resulting individual needs remained subordinated [10]. Other studies give an overview of how patients with lung cancer score their way of thinking using illness perception questionnaires; however this does not explain the underlying reasons [18–19]. Two qualitative studies focused on the manifest perceptions and perspectives of patients with lung cancer and advanced lung diseases in general, respectively [20, 21]. Regarding the diagnostic procedure, patients are either shocked or relieved, and some even resign themselves. Patients were informed and understood the diagnosis and prognosis, and focused on death and life expectancy, accompanied by a feeling of being swamped with and guided by disease issues. Other studies, which examined needs comprehensively and involved the patient perspective, did not, however, specify lung diseases [22–26].

To date, only little is known about the latent patients’ perspective and experiences of what it means to live with advanced lung cancer, and their resulting particular care needs at the end of life. In this study we understand an individual need not only as a conscious individual demand, but also as an unconscious issue that cannot always be verbalised [15–17]. Regarding the the serious symptoms, and their latent effects, lung cancer must be seen as a tremendous challenge for patients as well as relatives, family carers, and healthcare providers. In order to be able to guarantee patient-centred care of a high quality, in-depth knowledge of the patient’s individual needs is a prerequisite [15,16]. Against this background, the aim of this study was to gain a deep insight into patients’ experiences of living with advanced lung cancer and their individual needs.

## Materials and methods

### Design

The study presented in this paper was part of a larger qualitative longitudinal study covering a twelve-month observation period with four survey times (t_0_-t_3_). Since the design of the study is described in detail in the study protocol [26], only the main aspects are briefly outlined in this paper. The results presented in this article refer to the initial interviews (t_0_), because only in the baseline interviews were the patients asked to narrate their individual medical history and related illness experiences. The later interviews referred more to what has happened since the previous interview and to issues of the interview guide. The medical history is important to understand the ‘subjective-individual’ perspective of a person and allows an in-depth insight into the unconscious and nonverbal level of experiences. Interviews were conducted face-to-face at the patients’ homes (n = 12) or at the infusion clinic (n = 5) according to individual preferences. In the infusion clinic a pleasant and comfortable extra room was available, where the patients and interviewer were alone and undisturbed. Inclusion criteria were diagnoses of a Stage IIIB or IV (TNM system) small-cell or non-small-cell lung carcinoma. These stages foresee a palliative therapeutic approach without operative interventions [13]. Relatives were not explicitly invited to be present, but if both the patient and the relative agreed, relatives’ attendance and narratives were possible. The sample size corresponds to the sample size common in qualitative research [27]. In order to ensure the accuracy of the written English in this article, the manuscript was revised by a native speaker.

### Ethical considerations

The Ethics Committee of Hanover Medical School (Registration No.: 5896) and the University Medical Centre Göttingen (Registration No.: 19/11/12) approved the study. Patients with lung cancer received written information about participating in an interview in the study. This invitation stated that data would be pseudonymised and that identification by third parties would not be possible. It explained that participation is voluntary, and that the participating patients could also withdraw from participating at any time without giving a reason.

### Data collection

For data collection (2013–2014) we chose an open qualitative design which emphasises the subjective relevancies, structure and phenomena of a person’s lifeworld. To explore people’s subjective experiences is a prerequisite because they shape their perspectives within their lifeworld, which in turn influence their daily actions and needs. This process, however, is often unconscious and therefore not explicable. Social phenomena are explored to gain access to these experiences and needs. For that reason, appropriate research methods are needed to obtain access to the unconscious. In order to derive a deep understanding of the disease’s development in advanced lung cancer and the resulting individual needs, Grounded Theory was chosen to answer the research questions. Grounded Theory is based on a constant interchange between theory and empirical research to develop a deeper understanding of the experiences and individual needs of a patient with lung cancer [28,29]. The principles of Grounded Theory guided the research process, which is why we aimed at conducting theoretical sampling [28]. Aspects of sampling, for example, were changing needs within the course of the disease or needs depending on the place of living (rural or urban). Field access was provided by the pulmonary departments of two university hospitals and a specialist lung clinic in Germany. HS explained the study to all participants both verbally and in writing, and they each gave their written informed consent prior to the interview. The face-to-face interviews were introduced using an open question about the individual illness story, in order to evoke narrations along the patients’ relevancies and priorities. Additionally, an interview guide was used, focusing on care-related issues such as daily practical experiences, current problems, communication/information needs, and suggestions/wishes. For details of the interview guide, see the published study protocol [28]. After asking immanent questions based on the narration, the interviews were continued using an interview guide, with a focus on individual and illness-related needs and the current care situation, also using a narrative interview technique. No questions addressing the relatives’ perspective were prepared in advance. HS (Master of Science in Nursing (MScN) and doctoral candidate during the study) conducted the interviews, and was trained and supervised by GM (Ph.D., Master of Arts in Sociology (M.A.), highly experienced in qualitative research). Neither of them was involved in the patients’ care. The researcher did not give any special information about herself to the participants. Interviews lasted from 45 to 120 minutes, the average conversation duration was 63 minutes. All interviews were audio recorded and transcribed verbatim and subsequently checked for accuracy, but not returned to the participants. The statements made by the relatives were also transcribed and considered in the interpretive analysis in the context of the patients’ narrations. Field notes were made during and immediately after the interview. Data collection was finished when no new issues could be derived from analysis (data saturation).

### Data analysis

Analysis was conducted by two researchers (HS, GM) using Grounded Theory following Strauss and Corbin [28–30]. HS and GM conducted the analysis and repeatedly discussed the findings. HS wrote the first draft. Categories were derived from the data following abductive reasoning. Open coding and axial coding were conducted as an iterative process; in the last step, selective coding, the core phenomenon of patients’ experiences was worked out and all other main categories could be arranged according to the coding paradigm, which was introduced by Glaser and Strauss [30] and further developed by Strauss and Corbin. [28] The coding paradigm is subdivided into: causal conditions, action strategies, consequences, and closer and wider context (intervening condition and context) around a core phenomenon (core category) [28].

To ensure quality, the analysis process was embedded in the regular scientific and practical exchange within the interdisciplinary working group [26], where individual interview passages were regularly presented and discussed. MAXQDA 11 was used for data management.

The article fully adheres to the COREQ guidelines.

## Results

In all, 25 patients fulfilled the inclusion criteria, of which 17 persons with advanced lung cancer were willing to take part and were included in the study (14 men, three women, average age 62 years). The main reason for refusal was a lack of time due to time-consuming therapy. Interviews were conducted once, mainly in the clinic while awaiting chemotherapy. In nine cases, relatives were present. The sample is described in Table 1.

**Table 1:** Characteristics of the participants.

	Number of patients m/f
**Sample size**	14/3
**Age**	
45–64 years	5/2
65–78 years	9/1
**Residential area**	
urban (> 100,000 inhabitants)	5/3
suburban (town < 25,000 inhabitants)	8/1
**Classification of tumour (TNM Staging System)**	
Lung carcinoma stage III B	5/2
Lung carcinoma stage IV	9/1
**Histology**	
Squamous	3/1
Adeno	11/2
**Treatment modality**	
Chemotherapy	14/3
Radiation therapy	7/2
**Smoker status**	
Smoker	4/1
Ex-smoker	8/1
Never smoked	2/1
**Place of treatment**	
In-patient	6/1
Outpatient	8/2

**Fig 1:**
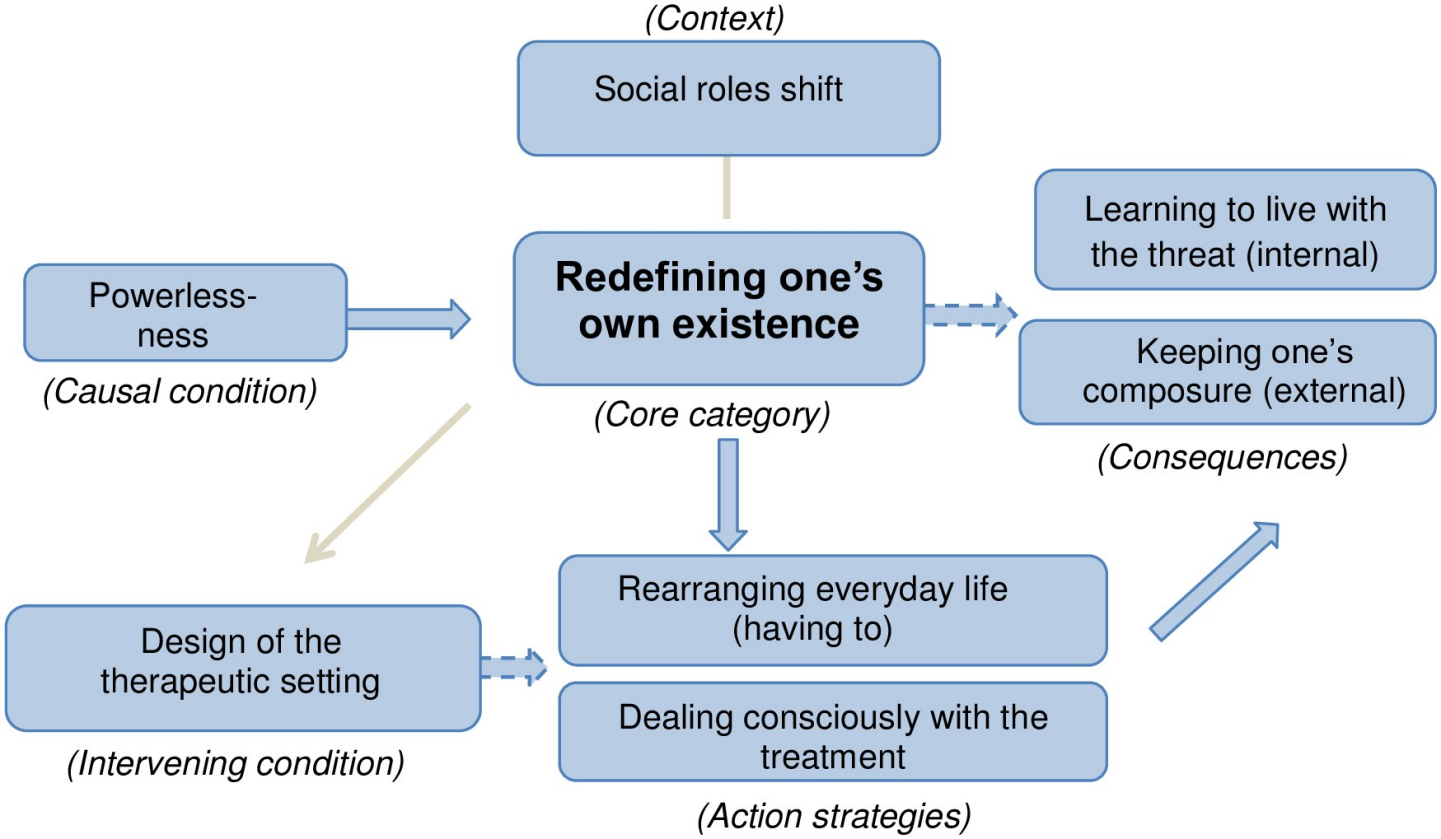
How patients experience life with lung cancer.

According to the coding paradigm we identified a core phenomenon and other main categories (causal condition, action strategies, consequences, and closer and wider context) The relationship of the core phenomenon to the other categories is shown in Fig 1 and will be presented in detail below [28,30].

### Redefining one’s own existence

The analysis shows that the experience of lung cancer is accompanied by the feeling of having to *redefine one’s own existence* (core phenomenon). This means that the disease lung cancer seems to assume a presence in the life of the afflicted in that it determines *one’s own sense of being*. Upon receiving the diagnosis, patients are confused about their former life, as also described by Yardley et al. [20]. The main cause of this phenomenon would appear to be above all the *powerlessness* perceived immediately after diagnosis, which shocks all previous life and experiences. Therefore, patients develop (action) strategies in order to cope with the disease-related challenges. One of these action strategies is the *rearrangement of everyday life*, which means that patients create new routines that seem to convey a feeling of security. Another strategy is the *individual way of dealing consciously with the treatments*, whereby the ability to act is regained by self-initiative. These action strategies are, however, encouraged or inhibited by structural conditions. Analysis revealed that the existing structural conditions of the *therapeutic setting* and its *type of design* are intervening conditions. Both the action strategies and the core phenomenon lead to consequences that appear to be evident in the experience of lung cancer patients in two different areas. These are patients being forced to *live with the threat*, yet nevertheless endeavouring to *keep their composure* externally. Firstly, patients deal with the threat to life posed by the disease and try to live with the threat inwardly. Secondly, they are in contact with their social and therapeutic environment, always endeavouring to maintain an attitude in order to signal a certain physical and psychological strength outwardly. These options for patients’ action and interaction are encouraged or inhibited by a context that manifests itself in the social and cultural environment in this analysis. The entire illness experience is codetermined contextually by the *role shift in the social fabric*, which begins at the time of diagnosis. The categories presented here, and the links between them, are shown in Fig 1 and will be presented in detail in the following sections.

### Powerlessness

Already the suspicion of a lung cancer diagnosis appears to trigger the feeling of powerlessness and helplessness in afflicted persons, especially when the diagnosis, as in the sample of this study, is made from a state of subjective good health.

“Everyone in my family then told me to go to the doctor, but not because of such a piffling cough [pause] I thought they had all gone mad [pause] so I inhaled a lot at home with my PARI BOY [pause] and when it didn’t subside I went to the doctor a n d then I lay there on the couch well-tanned looking healthy and the doctor looked at me looked at the x-ray looked at me again [tearfully] he couldn’t even believe himself how sick I am”

(Ms G., 53 years)

This helplessness is consolidated upon the confirmation of the diagnosis, whereby passivity arises, which is perceived as powerlessness. This passivity tends to mean that patients follow oncological treatment paths unconditionally, without considering their own needs or asking subsequent questions. The focus of these patients lies solely at the action level, i.e. on the tumour therapy.

" […] the first chemo was very bad, I thought they want to kill me here, I could not walk for five days and it hurt like crazy of course the hair fell out […] but that did not matter ((very loudly)) I didn’t give a shit about the hair"

(Ms. E., 61 years)

The psychosocial challenges of the patients and the change to their sense of being, with its associated individual problems and needs, are not sufficiently recognised in patients’ perception, either in the professional therapeutic setting or by relatives and friends:

“[…] on New Year’s Eve a friend of ours danced with me although he had tonsillitis that he had not told me about out of pure selfishness because he desperately wanted to see me [pause] and while dancing, he blew all his germs into my face and on New Year’s afternoon it started I also got a fever I also got a sore throat […] and I was out for the count could no longer walk or anything and I looked like death warmed up my husband had to drive me back to A&E at the M-hospital […] I spent a whole day there […] and then I cried my eyes out because the chemo was postponed I could not get the chemo on the third of January just because of this selfish friend […] and not getting the chemo that’s the only thing [tearful] I have”

(Mrs G., 53 years)

At the same time however, the feeling of powerlessness continues to increase due to the physical suffering experienced during palliative tumour treatment and the associated symptoms:

“I do perceive the chemotherapies to be very stressful [pause] the further back they are the better”

(Mr K., 55 years)

“Not [being able] to plan that is what really annoys me I am not actually a planning freak but I do rely a lot on my diary I have to say so [not understandable] until then and until then and that’s roughly how things should be and those are things […] it’s not because of me there is simply nothing I can do […] and that I don’t even know where things are headed what will happen [.] that’s the point I mean okay when I found out last week okay chemo let’s let things be and where I thought oh actually good now again you have not even five days where you feel sh- or not so well but on the other hand it is also a time when nothing happens and then Professor B. says I also want us to get going”

(Mr K., 45 years)

### Rearranging everyday life (having to)

Powerlessness in the context of oncological treatment can lead to patients seeking some certainty of action by asserting control over their actions. For example, a patient may take over the management of their own treatment by discharging themselves from treatment contrary to medical advice, undertaking changes to medication without consulting a doctor or nurse, or cancelling planned therapeutic measures without reason.

Interviewer: ((Looks at patients’ medication given unsolicited by the patient to the interviewer)) “Do you sometimes have swollen legs?”

Patient: “Oedemas […] I do not even take it anymore”

Interviewer: “[…] You’ve discontinued the drugs of your own volition […]”

Patient: “Yes yes”

Interviewer: “Did you discuss it with the doctor”

Patient: “No if I feel better if I do not want to take them then I don’t "

(Mr. W., 74 years)

Another strategy is to take a mental and emotional ‘break’ from the life-dominating disease by going on holiday with family or friends. Shifting the focus to the social area conveys a feeling of self-determination:

“[…] suddenly […] it started this infection […] then I was afraid that we would not be able to fly […] and beforehand I felt super I can no longer rely on that […] tomorrow I can suddenly feel unwell […] but we travelled there […] we were both scared […] that I would catch something there […] but it didn’t happen on the day we arrived the sun shone […] I felt like I was healthy […] and I truly forgot that I am ill.”

(Ms G., 53 years).

In addition, there is a great need to reintegrate into everyday life the routines that have had to be abandoned by the patient due to time-consuming treatment algorithms and severe symptoms resulting from side effects.

" […] that you have just this appointment every three weeks with of course GP appointments in-between and some check-ups and stuff [pause] ehm they make it impossible for me to even think of ehm work at the moment […] or of being able to resume working [pause] ehm and talk about anything and everything with colleagues or stuff like that"

(Mr. H., 51 years)

This is done, for example, by trying to reanimate old structures by adapting the previous intensity of everyday life to the current reduced capability (continuing to work with greatly reduced working hours). Implementing the need to continue working is a form of reinstating control that was initially lost with the sense of powerlessness. The necessity of rearranging everyday life is accompanied by patients becoming aware of their stress-triggering behavioural patterns and setting new priorities in order to counteract further treatment-related stress. This strategy makes it possible to implement a new (everyday) normality that is also compatible in terms of time with some treatment algorithms:

“I am a managing partner in this GmbH […] [and] have a lot of customer contact […] I miss that […] just the contact […] with other people is very important to me and even if it is by telephone you are in the midst of life and that also distracts you and I don’t let things affect me so much anymore anyway not like I possibly did before at times […] I don’t allow myself to get annoyed and that makes things easier […]”

(Mr K., 45 years)

### Dealing consciously with the treatment

The disease and the tumour treatment take on a central importance in patients’ everyday experience. This creates the need above all to *deal with the treatment consciously*, in order to be able to adapt to this new life situation. However, in the study patients stated that there was a lack of transparency in the therapy process, which restricts this subjectively necessary strategy for coping with the disease. Consequently, patients carry out their own research and are confronted with a lot of unfiltered information, e.g. from the internet.

“Well and then I give everything to my son he knows how to handle the internet and he prints everything out and so I read about it [pause] and when I’m with the oncologist next time, then I say ((taps on paper)) here it is written down and why don’t you treat me with that”

(Mr H., 77 years)

“[…] I’m always seen only as part of a statistic [not understandable] and then I think that there is always some part [pause] small part where it was different and you know I am a jurist so I keep on with my research to prove the opposite.”

(Mr K., 55 years)

Since it would appear that there is insufficient necessary clarification from doctors and nurses, treatment and care options are unclear, which can lead to dissatisfaction and renewed insecurity on the part of patients.

“[…] and nutritionally nothing happened either […] not in the O-hospital and not even in the M-hospital either [pause] according to the principle oh eat whatever you like and even psychological or complementary medicine no one tells you anything according to the principle you should try this or that or the physicians only see the chemotherapy and maybe their radiation and point out a surgical intervention if this is possible […]”

(Ms G., 53 years)

### Configuration of the therapeutic setting

The therapeutic setting was found to be an influencing factor with regard to coping with illness. In this phase of the illness, patients see palliative tumour therapy as the only treatment option. Starting from the medical consultation, patients have the impression that oncological therapy in particular can lengthen life, while palliative medical treatment does not.

"Palliative care, according to my definition, means that a curative approach is no longer possible and no longer desired […] so that you only accompany the patient to death […] ehm I emphasise more the negative side for me in it that it’s not curative anymore [pause] that you are finally shunned by conventional medicine [pause] I provoke I know that […] but this is what happens [pause] no one is worried anymore about whether something can be done somehow.”

(Mr K., 55 years)

For this reason, patients submit unquestioningly to the tumour therapy-controlled medical treatment algorithm, without considering possible additional therapy approaches. Analysis showed that time-consuming palliative tumour therapy creates deep trust in the professional competence of the attending physician, so that the necessary decisions of the patient can be greatly influenced by the statements of the doctor—even if other medical opinions deviate from that of the attending physician:

“[after the] induction chemotherapy […] it was checked again and Dr. (oncologist) said it had not grown […] so I should then go for radiation therapy which I did with Professor (radiation therapist) and there […] he said to me it […] looks as if the lung had got larger after all […] back to Dr. (oncologist) he said again […] no that is not the case that can be measured exactly […] and when I asked he replied […] if we had not done the chemo […] then we would surely have seen something in at least 6 weeks […] that is what he assumes […] and thus the next stage has been reached as far as I am concerned or as far as Dr. (oncologist) is concerned”

(Mr K., 45 years)

From the patients’ perspective, therefore, a sensitive, patient-centred and transparent communication style by doctors (e.g. time and the feeling of honesty and openness towards other therapy options), as well as regard for a patient’s own decision-making capacity (e.g. the feeling of being treated on equal terms) are essential for the *configuration of the therapeutic setting*, in order to feel well taken care of, and to promote acceptance of the therapy.

“[…] I just didn’t think that it would hit me so fiercely because nobody told (me) beforehand […] so I thought oh well chemo what’s going on here second course pff third course pff yes at some stage the bludgeon will come and that will be that yes Dr. (pneumologist) said that anyway he said ehm your values they constantly fluctuate let’s say like now [pause] let’s say last week I was here and they were still acceptable for continuing chemo now one week [later] yes it’s always like that he said of course you got both that can be explained by a [pause] I say alright and therefore now also probably the Tazewa in the meantime because it must have been clear to (name of oncologist) when he said we’ll take a break after that and then two more courses of chemo that was […] now we first have to his ehm immune system first has to sort itself out, yeah, so in that respect it makes sense, yeah, because he had said in the meantime which I never understood ((incomprehensible)) we’ll take a break and then two courses I said why if the tablets are working but that all seems to be part of a large plan that the doctors have”

(Mr. K., 52 years)

The positive or negative feeling that patients with lung cancer get from the configuration of the therapeutic setting is an influencing factor in how well or how poorly patients cope with the action strategy *to rearrange their everyday life*, and *to deal with the treatment consciously*.

### Learning to live with the threat and keeping one’s composure

The strategies developed from the necessity to redefine one’s sense of being in order to cope with powerlessness (*rearranging everyday life*, *dealing consciously with the treatment)* lead to behavioural consequences with a care-relevant impact: Internally, patients go through a process in which they must *learn to live with the threat (to life)*, to accept the severity of the disease and to cope with fears about the process of dying:

“[…] the illness in my case I think of it like a sore throat or a small flu to this day I can’t get it into my head that I have cancer and perhaps that is what keeps me the way I am”

(Mr T., 71 years)

In connection with this self-contradictory experience, it can happen that patients increasingly express a death wish in different ways, possibly with the help of the doctor, which apparently arises from the worry about increasing powerlessness, with a loss of self-determination. Such feelings appear to intensify due to the severe physical side effects during the treatment cycles, but decline during the breaks in therapy with the reduction in symptoms:

“[…] we do not determine the time ((takes a deep breath)) […] unless they say to me Ms E. I have a suggestion for you […] I’ll prepare an injection and then you’re gone […] I would say […] let’s do it […] because I am afraid of pain or of wasting away or being attached to some tubes despite everything or [.] afraid of experiencing death”

(Ms E., 69 years)

“[…] I wanted to be able to end my own life if I want [pause] not actively myself but through others [pause] passive insofar as they give me the means by which to do it”

(Mr W., 74 years)

In strong contrast to this internal experience, patients interacting with other persons, e.g. also with therapists, have the need to *keep their composure* externally, which contributes to concealing their physical and in particular their psychological symptoms:

Interviewer: "Ehm [pause] were you given something for the nausea?”

Patient: ((negative)) “Ehmehm […]”

Interviewer: “Nah [.] was it offered to you”

[…]

Patient: “No […] I didn’t even mention it […] otherwise they would have kept me here so […] yeah, the customers don’t even ask if I’m fine, no no so if they do I’m not sick, it was nothing, nah, I tolerated it well yes”

Interviewer: “But you haven’t mentioned the loss of appetite or nausea”

Patient: “No, no […]"

(Mr. L., 63 years)

“[…] I notice now for example [.] we are chatting [pause] that the tears are flowing, [nobody] has ever seen that with me ever […] it’s sometimes possibly [pause] false male pride […] [pause] [tearful] men don’t cry”

(Mr Ü., 54 years)

### Social roles shift

The action strategies and interactional options of patients explained above are supported or restricted by means of a context that is manifested in the social environment of people with lung cancer. Over the course of the illness, the social roles of the participants in our study changed, this is why it was possible that, within a very short period, previously independent people became patients with a great need for help and support. Analysis suggests, that, among male patients in particular, this is associated with a change, e.g. from being the ‘family provider’ to a person in need of care, which is not easy to accept. The well-intended care offered by relatives and friends is acknowledged, but not always accepted, even though the disease dominates and determines the perception of the (social) existence. Thus the role of patient is what is primarily perceived, and not the previous role, for example as spouse, parent or friend:

“[in the] private environment family environment [.] great concern […] [pause] some are uncertain [pause] [.] very many offers of help naturally from my group of friends although they can’t do anything either [pause] […] I don’t need anyone to steer my rollator [pause] I can still manage alone”

(Mr H., 56 years)

Nevertheless, the shift in roles can also cause patients to experience positive feelings, and not only stress, since the diagnosis can lead to increased psychosocial cohesion within the family and with friends.

" […] the parents-in-law [are] very important ehm there have been some developments well they have changed in a positive way- (we once were) kind at daggers dawn typically [pause] no not them again and ehm when they also heard of that then then they were standing on our doorstep and ehm have absolutely they were riding along to the M-hospital anyways ehm my wife was like ‘well can’t you come along’ and this and that well they have really done everything em taken care of things around the house like I don’t know come over here cutting the trees in the sense well that I underwent chemo therapy and so on well and I mean well then I truly realised that that I have done wrong to those people over a long period of time well this has really flip-flopped"

(Mr K., 46 years)

## Discussion

This study suggests that lung cancer can be perceived as a highly threatening disease which determines one’s own sense of being. The diagnosis can cause a feeling of powerlessness leading to ‘information passivity’ and the unconditional acceptance of aggressive tumour treatment. Patients are considerably impaired by potential side effects, so that daily routines are omitted. After a period of powerlessness, they feel the need to get back to some everyday routines (e.g. carry out their profession). Analysis showed further, that patients demonstrate ambivalent behaviour. On the one hand, they realise the rapidly advancing progression of their disease, which is based on the recurring experience of physical weakness, followed by increased fear of experiencing a painful dying process. On the other hand, they continue to foster the hope of being healed and misunderstand their physicians’ statements and the aim of their therapy (e.g. curative instead of palliative). It is difficult for physicians to detect these misunderstandings because patients try to retain their composure when interacting with their family, friends, and carers to uphold the pretence of their physical and psychological strength. As a consequence of being able to deal with their daily life, the patients in this sample hid their high level of physical and psychological stress, with, though, the consequence that psychosocial care was not provided.

### Relevance of psychological support

A consequence of living with advanced lung cancer, is that one’s own existence must be redefined (core phenomenon, see Fig 1) for personal reasons (e.g. fear) and external reasons (e.g. therapies). A central cause is the threat to life that is associated directly with the diagnosis and leads to a high stress potential, as also shown by other studies [8–10,20,21]. Upon diagnosis, psychological stress and powerlessness are triggered and, as shown by a large-scale cohort study in Sweden, can—when ignored—lead to an increased suicide rate or a higher death rate due to stress-related cardiovascular causes [31]. Professional medical personnel in all settings (in-patient, outpatient, specialised, general), should pay careful attention and recognise warning signals for crisis situations associated with any death wish expressed by lung cancer patients. The lack of communication within the health professionals and between patients and carers, [32] require special attention to good communication. In Germany, general practitioners (GPs) play a key role, since they generally have provided medical support to the patient for the longest time. However, a systematic review of references to GPs in oncological guidelines has shown that in Germany in particular, the GP is rarely involved in the care programme of oncological patients in the initial treatment phase [32]. This could explain why, despite the results of many other studies [9–11,20–25] and recommendations by guidelines [13–16], the involvement of psycho-oncological support for (lung) cancer patients, as shown in this study, is rarely used. A further possible reason is that patients often reject psychotherapeutic support, since the fear of dying and the accompanying process are not seen as a type of stress that requires professional psychological support. The need for specific provision of psychological support [9–11,2–25] rather than a vague offer, shows clearly the necessity to revise the previously practised access to psycho-oncological measures. Villalobos et al. [33] suggest the implantation of a longitudinal communication approach, to promote flexibility for respecting the individuality of patients with lung cancer, as one way of improvement.

### Ambivalence of palliative tumour therapy

Although the increased average survival rate due to palliative tumour treatment is proven in studies, the equally important assessment of quality of life, perceptions, and the existing individual disease-specific patients’ needs are insufficiently reflected [34]. Palliative tumour treatment implies a paradox as also shown in other studies: patients’ increased suffering and patients’ wish to receive it [21]. The tumour treatment may provide psychological amelioration by conveying hope increases suffering due to the associated physical stress at the same time. These physical effects also impede the implementation of the distinct need for *everyday (re-) arrangement*, which in turn causes suffering that is more psychological. In severe cases, this may prompt a wish to die, with the help of a doctor. Fegg et al. [35] explained that the termination of palliative tumour treatment due to a poor general condition of health leads to a reduced quality of life, which in turn seems to be strongly related to hope. Despite the general knowledge about lung cancer, these findings show that facing death may still lead to profound communicative misunderstandings, as was already described by Yardley et al. almost 20 years ago [20].

A systematic review of the relevance of hope to dying people by Broadhurst and Harrington [36] pointed out that seriously ill patients shift their attitude to their life situation and their will to survive during the course of their illness: From the initial focus on accepting the bad prognosis to hope for a cure, and at least to a peaceful dying process. This explains why the mediation of hope is particularly beneficial and contributes to an increased quality of life [36,37].

### Multidimensional design of the therapy

As shown in this study, disease related experiences should be considered in order to capture interpersonal perspectives and to determine individual needs. In the German S3 guidelines, recommendations are made that orient the treatment of progressive diseases to patients’ individual needs [13–16]. Regarding the patients’ individual necessity of having to redefine their own existence, the biopsychosocial care approach is appropriate, since this includes both physical and psychosocial areas. As already shown by Goodridge et al. and Yoong et al. [6,38], it also became clear in this study that the physical dimension was addressed by means of tumour-specific and medicinal supportive measures. However, palliative care, psychosocial or psycho-oncological care and nursing care remain subordinate during tumour treatment. Yet the relevance of a multidimensional care approach is already recommended [13–16]. It is known that palliative care in addition to tumour-specific therapies can improve quality of life, reduce distress and depression [11], strengthen coping capabilities and an understanding of the disease and prognostic awareness [38], and may possibly increase the median survival rate [39]. The necessity to close the gap in this previously neglected area of care thus becomes all the clearer.

### Limitations

A limitation of this study is that patients have been recruited only in one federal state in Germany, and only in two university hospitals and cooperating hospitals. Because of the specific principle of supramaximal care in university hospitals, the patients of our sample might differ from other patients. A second limitation is that this study did not follow a recruiting strategy covering the outpatient setting, e.g. general practitioners. We assume that patients diagnosed with lung cancer strengthen their contact to their oncologist, and contact their general practitioner only in the final stage of dying. It cannot be ruled out that patients and their relatives recruited in other outpatient settings might have different experiences and needs. The fact that relatives were present in some interviews could be seen as strength or limitation of data collection. However, it must be considered that single or dyadic interviews could produce different but also valuable insights. [40]

Although this study intended to cover the relevance of gender specific differences, only three women, compared to 14 men, agreed to participate. Reason for refusal was lack of interest. Analysis showed that gender might be an issue in patients’ illness experiences and needs, but further research is necessary to deepen these aspects. Covering only 17 patients in total, the sample was nevertheless sufficient to reach data saturation, meaning that the findings should be generalisable. The results provide a deep insight into patients’ disease-specific experiences and needs, made possible because the informants were willing to talk frankly and in detail about their life and feelings.

## Conclusions

The findings of this study show the necessity of a high level of teamwork in multi-professional teams, including good communication, to be able to meet the specific communication needs of patients facing death. An interdisciplinary and multi-sectoral cooperation including primary care, specialists and palliative carer right after diagnosis should be mandatory in order to meet the patients’ needs and perspectives. Trained volunteers can offer repeated dialogues and have time to listen to the patient, which might counter the situation that 1) patients do not always communicate their individual needs, even when asked, and 2) sometimes are not aware of their needs, because they may be subconscious. This would require, however, close communication between volunteers or hospice services and the healthcare provider.

It can further be helpful to integrate assessments at an early stage across all professions into the multi-professional treatment to display the needs for support much more clearly from different professional perspectives. Thus, further research is urgently needed on whether the expansion of existing multi-professionally applicable assessments (e.g. Integrated Palliative Care Outcome Scale, IPOS) can facilitate the early detection of individual patient needs.

To focus the specific psychosocial needs of patients who receive their chemotherapy in hospital, followed by a three-week rest period, there should be more support in qualification programmes to increase the function of medical, nursing and psychosocial care for these outpatients.

## Supporting information

S1 Table Interview guide English. (PDF)

S2 Table Interview guide German. (PDF)
