# Supplementary material for: “I can’t get it into my head that I have cancer…”—A qualitative interview study on needs of patients with lung cancer
Source: PLoS One. 2019 May 14;14(5):e0216778. doi: 10.1371/journal.pone.0216778 (PMC6516640; doi:10.1371/journal.pone.0216778)
Supplement: S1 Table — (PDF) [file pone.0216778.s001.pdf]

**Interview guide for serial interviews with patients***
**Introduction and opening question**<br>• How do you feel today? Is there anything you want to tell before we start the interview?<br>• We are interested in individual experiences of people diagnosed with severe lung diseases and aim to contribute to improve care related needs for these patients.<br>*Invitation to tell*: Please tell me what happened, when the disease was diagnosed and what happened in your life since that time. May be you could elaborate on all details that seems relevant for you. Please take as much time as you want.<br>*Stage direction*: While you tell, I will restrain requesting, but listen to your story. I will just make some notes and come back to them later. We have sufficient time. If you need a pause, please tell me.
**Additional questions**<br>*(as long as they were not yet mentioned, or to deepen the issue)*<br>**Daily practical experiences, possible problems, and structural support available**<br>• Please tell me a typical daily routine<br>• Please tell me a typical weekly routine, including weekend<br>• Thinking about your daily or weekly routine: where do you need daily support? Do you get this support? What kind of changing do you wish to have? May be you remember some situations you could tell me?<br><u>Additional</u> (if required)<br>• What kind of support do you receive presently?

- How was your present support be arranged? Did you know, what kind of support exist for a person suffering from a severe lung disease? Did anybody help you with that?
- Please think: What kind of further support could help you?

Current problems and issues

Symptoms, discomfort

- What kind of symptoms, discomfort or concomitant effects of the disease or the therapy are currently problematic for you? How are they treated?
- What kind of examination and treatment do you currently receive and what are they for?
- What do you feel, to what extent these examinations and treatment impair your daily life? (How do you perceive this?)

Social contacts

- Please tell me something about your social contacts, your relationship to your friends and family. How were they before you were diagnosed and how are they now? May be something has been changing over time? Please tell me single situations that stick in your mind.

Personal issues

- What personal issues currently bother you? (worries/fears)
- What are you doing to solve these issues? How did you get the idea to do this?
- When you look back: In the context of your disease, what did surprise you mostly?

Communication and information needs

- Do you remember the encounter, when the diagnosis was told you? What happened? How did the following initial clinical interviews rsp. intake interviews

with doctors and nurses or other involved personnel after diagnosis?<br>• With whom do you speak currently about your disease, prognosis and therapies? What happens during these encounters?<br>• What kind of experiences did you make in facilities that you hat to visit during your disease (hospitals, emergencies, rehab clinic, therapy center)? Please tell me about positive and/or negative experiences you have made.
**Suggestions/wishes**<br>• Please imagine optimal care: How should this be organized and constituted? Which wishes do you have? What should remain unchanged, what should be changed?
**Closing the interview**<br>• Is there anything you want to tell me, may be something that is important to you, and we didn't have talked about?<br>• How do you feel now? How did you experience the interview?

*This is a translated version of the German original for the purpose of publication.
