# Supplementary material for: “I can’t get it into my head that I have cancer…”—A qualitative interview study on needs of patients with lung cancer
Source: PLoS One. 2019 May 14;14(5):e0216778. doi: 10.1371/journal.pone.0216778 (PMC6516640; doi:10.1371/journal.pone.0216778)
Supplement: S2 Table — (PDF) [file pone.0216778.s002.pdf]

Table 2 Interviewleitfaden für Serieninterviews mit Patienten

Einleitung in das Interview und offene Eingangsfrage (Haupterzählung)

- Wie fühlen Sie sich heute? Gibt es etwas, das Sie erzählen möchten, bevor wir mit dem Interview anfangen?
- Wir sind ja an den Erfahrungen und Bedürfnissen von Menschen mit schweren Lungenerkrankungen interessiert und wollen dazu beitragen, dass die Behandlungssituation für diese Patientinnen und Patienten verbessert wird. *Erzählaufforderung*: Bitte erzählen Sie mir doch nun einmal, wie es war, als die Erkrankung bei Ihnen festgestellt wurde und was seitdem (in Ihrem Leben) passiert ist, bis heute. Es wäre schön, wenn Sie dabei auf alle Details eingehen würden, die Ihnen wichtig erscheinen. Sie können sich dabei so viel Zeit nehmen, wie möchten.
- *Regieanweisung*: Während Sie erzählen, werde ich mich mit Kommentaren oder Nachfragen zurückhalten und Ihnen ausreichend Zeit zum Erzählen geben. Ich werde mir nur ein paar Notizen machen und dann später darauf zurückkommen. Wir haben ausreichend Zeit. Falls Sie eine Pause brauchen, sagen Sie es bitte.

Ergänzender Leitfaden

(sofern diese Themen noch nicht angesprochen wurden, bzw. zur Vertiefung)

Praktische Probleme und strukturelle Unterstützung

- Bitte schildern Sie mir doch einmal einen typischen Tagesablauf
- Bitte schildern Sie mir nun, wie bei Ihnen eine typische Woche incl. Wochenende

verläuft.

- Wenn Sie noch einmal an ihren Tages- oder Wochenablauf denken: worin benötigen Sie im Alltag (am meisten) Unterstützung? Bekommen Sie diese Unterstützung? An welchen Stellen wünschen Sie sich eine Veränderung? Vielleicht erinnern Sie sich auch an einzelne Situationen, die sie mir schildern können?

Gegebenenfalls ergänzend:

- Welche Arten von Hilfe/Unterstützung erhalten Sie derzeit?
- Wie wurde ihre aktuelle Unterstützung/Hilfeleistung arrangiert? Wussten Sie, welche Möglichkeiten der Unterstützung es für Personen gibt, die wie Sie, eine schwerwiegende Erkrankung haben? Hat Ihnen dabei jemand geholfen?
- Wenn Sie einmal überlegen: Was könnten Sie aktuell darüber hinaus an Hilfestellungen/Unterstützung gut gebrauchen?

Aktuelle Probleme und Themen (physisch, psychosozial, spirituell) und zur Verfügung stehende Unterstützung

Symptome, Unwohlsein

- Welche Symptome, Beschwerden oder Begleiterscheinungen der Erkrankung oder der Therapie sind für Sie aktuell problematisch? Wie werden diese behandelt?
- Welche Untersuchungen und Behandlungen bekommen Sie derzeit und wofür sind diese?
- Inwieweit beeinträchtigen diese Untersuchungen und Behandlungen Ihr Leben?

(Wie empfinden Sie das?)<br><br>Soziale Kontakte<br><br>• Bitte erzählen Sie mir doch etwas über Ihre sozialen Kontakte, Beziehungen zu Freunden und zur Familie (Bezugsperson?). Wie waren sie vor der Erkrankung und wie sind sie jetzt. Vielleicht hat sich im Verlauf der Behandlung etwas verändert? Bitte gehen Sie auch auf einzelne Situationen ein, die Ihnen besonders im Gedächtnis geblieben sind.<br><br>Persönliche Themen<br><br>• Welche persönlichen Themen beschäftigen Sie aktuell? [Sorgen/Ängste]<br>• Was unternehmen Sie, um diese Themen für sich zu lösen? Wie sind Sie darauf gekommen?<br>• Wenn Sie zurückblicken: Was hat Sie im Zusammenhang mit Ihrer Krankheit am meisten überrascht?
**Kommunikation und Informationsbedarf**<br><br>• Erinnern Sie sich noch an das Gespräch, als Ihnen die Diagnose mitgeteilt wurde? Wie ist das abgelaufen? Wie verliefen die weiteren Erst- bzw. Aufnahmegespräche mit den Ärzten und dem medizinischen und pflegerischen Personal zu Beginn Ihrer Erkrankung?<br>• Mit wem sprechen Sie aktuell über ihre Erkrankung, Prognosen und Therapien? Wie verlaufen diese Gespräche?<br>• Welche Erfahrungen haben Sie in Bezug auf Einrichtungen die Sie im Verlauf Ihrer Erkrankung aufsuchen mussten (Krankenhäuser, Notaufnahme,

Anschlussheilbehandlungen, Rehabilitation, Therapiezentren) gemacht? [Bitte erzählen Sie einmal, welche positiven oder negativen Erfahrungen sie gemacht haben]
**Vorschläge/Empfehlungen**<br><br>• Wenn Sie sich jetzt einmal eine optimale Versorgung vorstellen: Wie sollte diese aussehen? Was wünschen Sie sich? Was sollte so bleiben, was sollte verändert werden?
**Interviewabschluss**<br><br>• Gibt es noch etwas, was sie ansprechen möchten, was ihnen wichtig ist, was wir aber bisher nicht besprochen haben?<br>• Wie geht es Ihnen jetzt? Wie war das Interview für Sie?
